# Functions and mechanisms of miR-650 in human diseases

**DOI:** 10.3389/fmolb.2026.1911030

**Published:** 2026-09-11

**Authors:** Qingmiao Shi, Chengran Gao, Chen Xue

**Affiliations:** 1 The First Affiliated Hospital, College of Clinical Medicine, Henan University of Science and Technology, Luoyang, China; 2 The First Affiliated Hospital of Zhengzhou University, Zhengzhou, China

**Keywords:** cancer, ceRNA, function, mechanism, miR-650, non-neoplastic disease

## Abstract

MicroRNAs (miRNAs) are endogenous, single-stranded, small non-coding RNAs that play central roles in gene expression regulation. Among them, miR-650 has received increasing attention over the past decade because it regulates cell proliferation, differentiation, apoptosis, metabolism, cell cycle control, immune responses, and development. miR-650 has been studied in both neoplastic and non-neoplastic disease contexts. This review summarizes the expression characteristics of miR-650 in human diseases and discusses its roles in disease initiation and progression, the pathways in which it participates, and the upstream and downstream regulatory mechanisms involved. We also discuss the potential clinical value of miR-650 in disease diagnosis and therapy. Defining shared pathways across diseases and disease-subtype-specific expression patterns within miR-650-related regulatory networks may help identify more precise therapeutic targets.

## 1 Introduction

**FIGURE 1:**
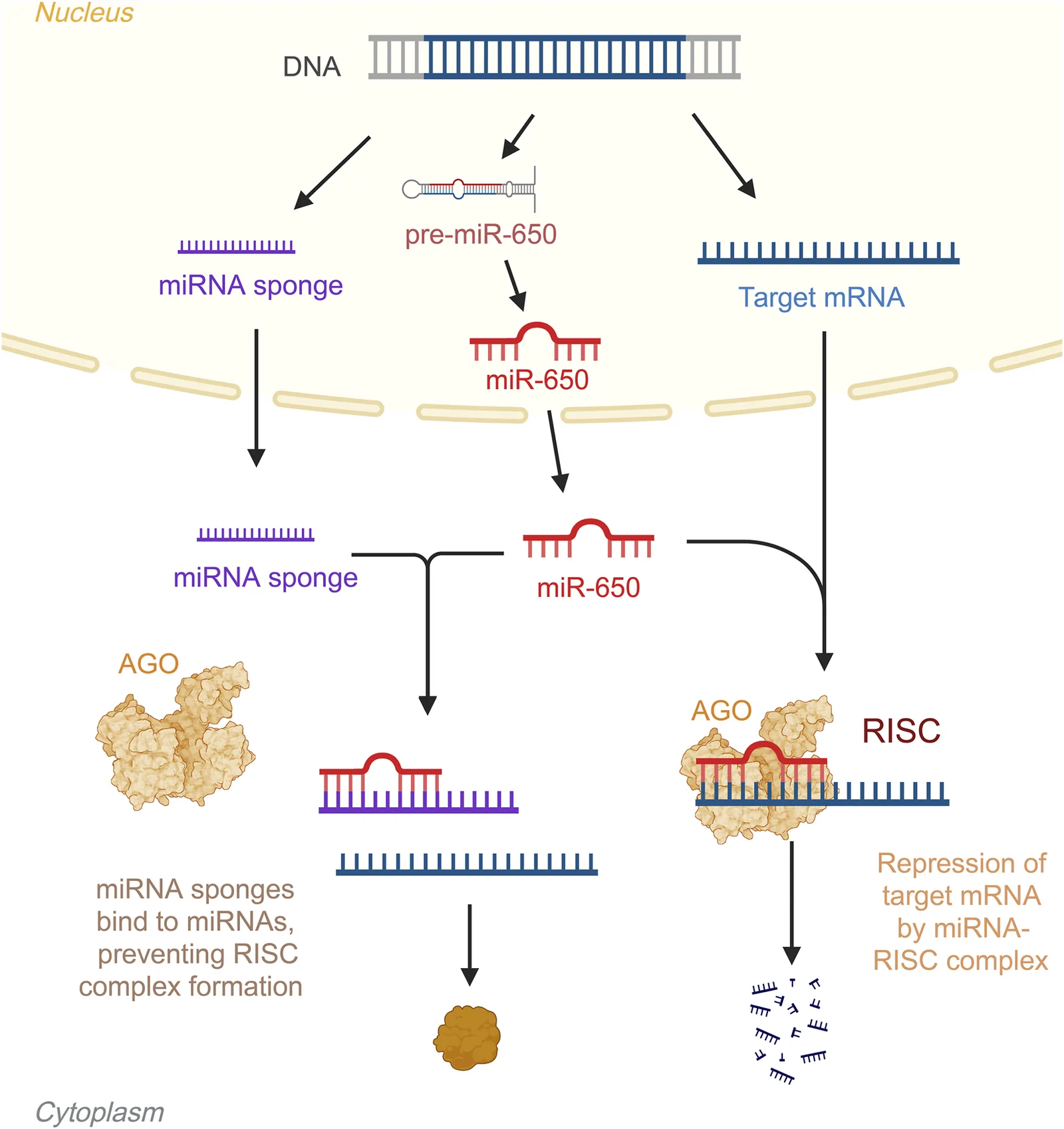
The regulation of the target mRNA by ceRNA-miR-650. In the absence of a miRNA sponge, miR-650 incorporates into the RISC, binds to the target mRNA, and degrades it. When miRNA sponges are present, they competitively bind miR-650, protecting the target mRNA and allowing its expression.

MicroRNAs (miRNAs) are endogenous, single-stranded, non-coding small RNAs that play central roles in gene expression regulation. They are typically 21–25 nucleotides long and belong to the family of short non-coding RNA (Su et al., 2025). miRNAs are transcribed from hairpin-shaped precursors and are widely present in animals, plants, and viruses, with a high degree of evolutionary conservation (Mortazavi et al., 2021). Since their discovery in 1993, approximately 2,000 miRNAs have been identified in the human genome (Wang, 2024). Mechanistically, miRNAs bind complementarily to the 3′ untranslated region (UTR) of target mRNAs and, through the RNA-induced silencing complex (RISC), induce mRNA degradation or translational repression. They can also be sequestered by upstream competing endogenous RNA (ceRNA) sponges, which relieves repression of downstream target genes (Figure 1) (Liu et al., 2022). miRNAs regulate a substantial proportion of human protein-coding genes and play important roles in diverse biological processes, including development, metabolism, immune regulation, and disease progression (Zheng et al., 2025).

miRNAs are involved in key biological processes, including cell proliferation, differentiation, apoptosis, metabolism, cell cycle regulation, immune responses, and development. Their expression is dysregulated in multiple tumors, including lung, adenocarcinoma and squamous cell carcinoma, where they may act as oncogenes or tumor suppressors and influence tumor initiation, progression, and metastasis. miRNAs are also closely associated with neurodegenerative, cardiovascular, autoimmune, and infectious diseases (Fattahi et al., 2023).

The disease-associated expression patterns and molecular mechanisms of miR-650 have not been systematically characterized. Growing evidence nevertheless links aberrant miR-650 expression to disease development. This review examines the potential clinical value of miR-650 in diagnosis and therapy. As a candidate diagnostic and therapeutic tool, miR-650 may serve as a disease biomarker or as a molecular basis for targeted intervention, although stronger experimental evidence is needed to define its mechanisms and support clinical translation.

The previous review mainly discussed the aberrant expression patterns, functions and potential mechanisms of miR-650 in various cancer tissues and cell lines. In contrast, this review integrates malignant and non-neoplastic diseases to identify shared regulatory architectures and disease-specific functional polarity. Particular attention is given to the ceRNA–miR-650–target network, recurrent downstream hubs such as ING4, LATS2 and AKT2, pathway crosstalk, contradictory expression patterns, and translational barriers. By comparing miR-650 with other multifunctional miRNAs and distinguishing preclinical evidence from clinically supported observations, this review provides a disease-crossing framework for understanding the biological and translational significance of miR-650.

## 2 Bioinformatic prediction of miR-650 target genes and associated pathways

MicroRNA-650 (miR-650) is a non-coding RNA closely associated with tumorigenesis and cancer progression. It is aberrantly expressed in multiple malignancies, including gastric cancer, non-small cell lung cancer, and glioma. A comprehensive definition of the miR-650 target-gene spectrum and its regulated signaling pathways is essential for understanding its pathogenic mechanisms. Bioinformatic prediction provides an efficient and systematic approach for this purpose. Current miRNA target prediction generally uses complementary algorithms, including TargetScan, miRDB, DIANA-microT, and miRWalk (Agarwal et al., 2015; Chen and Wang, 2020; Paraskevopoulou et al., 2013; Sticht et al., 2018). Identification of reliable miR-650 targets requires integration of computational prediction, experimental databases, disease-specific expression profiles, and functional validation. A typical workflow begins with prediction using multiple algorithms, followed by filtering through experimentally validated databases such as miRTarBase and TarBase. Candidate targets should then be integrated with transcriptomic datasets to identify disease-relevant genes exhibiting inverse correlation with miR-650 expression.To improve reliability, most studies use an intersection strategy, selecting genes predicted by at least two algorithms and cross-referencing them with experimentally validated databases such as miRTarBase and TarBase to construct high-confidence target sets (Huang et al., 2020; Karagkouni et al., 2018).

Several computational tools have been developed to predict miRNA targets; however, each platform has specific strengths and limitations. TargetScan emphasizes evolutionary conservation and seed matching, whereas miRDB incorporates machine-learning algorithms to improve prediction accuracy (Dweep et al., 2013). DIANA-microT evaluates multiple sequence and structural features, while miRWalk provides broad prediction coverage by integrating several algorithms (Dweep et al., 2013). Nevertheless, computational predictions alone are insufficient because false-positive predictions remain common. Therefore, predicted targets should be further supported by experimental evidence, including expression correlation analysis, luciferase reporter assays, miRNA mimic/inhibitor experiments, rescue assays, and ideally *in vivo* validation (Lin and Qiu, 2025; Militello et al., 2025; Pantazi and Zovoilis, 2013; Tomasello et al., 2019).

Studies integrating differential expression with target prediction can further refine candidate targets. For example, a breast cancer study used the cBioPortal database to analyze samples with miR-650 amplification (Amp-650). By combining target-prediction tools with a tumor-suppressor gene database, the study identified hundreds of downregulated genes and nominated STAT5A as a potential miR-650 target in aggressive, poorly differentiated breast cancer (López-Huerta and Fuentes-Pananá, 2020). For pathway enrichment analysis, researchers frequently use databases such as KEGG to functionally annotate predicted miR-650 targets. In Alzheimer’s disease, bioinformatic analysis predicted that miR-650 targets APOE, PSEN1, and CDK5, all of which are associated with AD pathogenesis (Lin et al., 2023). In Crohn’s disease, enrichment analysis of miR-650 targets identified the alanine, aspartate, and glutamate metabolism pathway (Steigleder et al., 2024). Together, these bioinformatic predictions align with experimental findings across multiple cancer types and support the view that miR-650 may function as a regulatory hub in tumor-associated signaling networks.

## 3 Functions and mechanisms of miR-650 in human cancer

Malignant tumors remain a major public health challenge worldwide. Recent estimates indicate that there were nearly 13.58 million new cancer cases worldwide in 2025, and the number is projected to reach 19.32 million by 2050 (Ward et al., 2026). Strengthening prevention-centered cancer strategies is therefore essential. With population aging, changing lifestyles, and environmental exposure, cancer incidence and mortality continue to rise. Although surgery, radiotherapy, chemotherapy, targeted therapy, and immunotherapy continue to advance, tumor heterogeneity, metastasis, and drug resistance remain major clinical challenges. Detailed investigation of the molecular mechanisms underlying tumor development and identification of new diagnostic markers and therapeutic targets are therefore scientifically and clinically important.

miR-650 illustrates this regulatory complexity. Recent studies indicate that miR-650 is aberrantly expressed in multiple malignant tumors, including colorectal, lung, gastric, breast, liver, and skin cancers, as well as glioma. However, miR-650 does not have inherent “oncogene” or “tumor suppressor gene” properties. Its biological functions are highly dependent on the dynamic interaction between the upstream regulatory network and the cellular microenvironment. From the upstream transcriptional regulation, processing and maturation, to the accessibility of downstream target mRNAs, the buffering effect of the competitive endogenous RNA (ceRNA) network, as well as the overall genetic background and disease progression stage of the host, every minor change in each link can reshape the functional output of miR-650. Therefore, the ultimate biological effect of miR-650 is essentially a multivariate function of cell type, target spectrum status, ceRNA network, genetic background, and disease stage (Su et al., 2022). This section reviews the regulatory mechanisms of miR-650 across cancer types, with emphasis on direct targets, upstream lncRNA regulatory axes, and related signaling pathways, to provide a basis for further basic research and clinical translation (Table 1).

**TABLE 1:** Expression and regulation of miR-650 in tumors.

Cancer type	miR-650 expression	Role of miR-650	Regulatory axis	Function	Reference
Colorectal cancer	/	Oncogene	miR-650/ING4	Proliferation, Migration, EMT, Skeleton rearrangement	(You et al., 2018)
	/	Oncogene	CpG hypermethylation + miR-650/NDRG2	Translation	(Feng et al., 2011)
	Downregulation	Tumor suppressor	miR-650/KISS1	Cell viability, Apoptosis; Resistance	(Valizadeh et al., 2023)
	/	Tumor suppressor	DICER1-AS1/miR-650/MAPK1	Cell growth, Proliferation	(Li et al., 2023)
	/	Tumor suppressor	MIR155HG/miR-650/ANXA2	Malignant phenotype, Polarization of M2 macrophages, Resistance	(Zhou et al., 2022a)
	/	Oncogene	MIR22HG, RP11-61113.3/miR-650/BTK, PDK4	/	(Liao et al., 2019)
	Downregulation	/	miR-650, miR-150-5p/NF-κB	/	(Slattery et al., 2018)
Lung cancer	Upregulation	Oncogene	miR-650/ING4/Wnt-1, β-catenin	Proliferation, Invasion Prognosis	(Tang et al., 2019)
	Upregulation	Oncogene	miR-650/ING4/Bcl-2, Bax	Prognostic, Apoptosis, Chemotherapy sensitivity	(Huang et al., 2013)
	Upregulation	Oncogene	CARD8-AS1/miR-650/Bax	Prognosis	(Ji et al., 2022)
	/	Tumor suppressor	APA → NIT2 evades inhibition by miR-650	Proliferation, Apoptosis, Growth, Malignant phenotype, Onset risk	(Xu et al., 2024)
Gastric cancer	Upregulation	Oncogene	miR-650/ING4	Proliferation, Tumor formation, Lymphatic metastasis	(Zhang et al., 2010)
	Upregulation	Oncogene	PBX1/miR-650/LATS2 (Hippo)	Proliferation, Migration, Invasion, Apoptosis	(Liu et al., 2021a)
	Upregulation	Oncogene	DICER1-AS1/miR-650/CSR1	Proliferation, Migration	(An et al., 2021)
	Upregulation	Oncogene	linc01105/miR-650/TCEA3	/	(Zhang et al., 2025)
Melanoma	Upregulation	Oncogene	miR-650/ING4	Invasion, Migration, Metastasis	(Chen et al., 2024)
	/	Tumor suppressor	ZFPM2-AS1/miR-650/NOTCH1	Proliferation, Migration, Apoptosis	(Liu et al., 2021b)
	/	Tumor suppressor	POU3F3/miR-650/MGMT	Resistance	(Wu et al., 2021)
Breast cancer	Upregulation	Oncogene	miR-650/STAT5A	EMT, Prognosis	(López-Huerta and Fuentes-Pananá, 2020)
	Upregulation	Oncogene	22q11.2 amplification/miR-650/ING4, NDRG2	EMT, Invasion, Cancer stem cell phenotypes	(Lango-Chavarría et al., 2017)
Glioma	Upregulation	Oncogene	/	Classification, KPS, overall survival	(Sun et al., 2013)
	Upregulation	Oncogene	NF-κB1/miR-650/RERG/RERG-PHLPP2 complex/AKT, ERK, NF-κB	Proliferation, Migration, Invasion, EMT, Autophagy, Apoptosis	(Jin et al., 2020)
HCC	Downregulation	Tumor suppressor	miR-650/TRAF4	Non-invasive urine markers	(Abdalla and Haj-Ahmad, 2012)
	Upregulation	Oncogene	B[a]P/miR-650/SOCS3/JAK-STAT3	Metastasis	(Ge et al., 2021)
	Upregulation	Oncogene	Axin1/miR-650	Proliferation, Migration, Invasion, EMT	(Qin et al., 2020)
	Downregulation(Drug treatment group)	Oncogene	NBR2/miR-650/TIMP3	Proliferation, Migration, Invasion	(Liang and Ke, 2022)
Prostate cancer	Upregulation	Oncogene	LINC01002/miR-650/FLNA	Proliferation, Migration	(Qian et al., 2023)
	Upregulation	Oncogene	miR-650/CSR1	Proliferation, Metastasis, Prognosis	(Zuo et al., 2015)
Ovarian cancer	Downregulation	Tumor suppressor	miR-650/KLF12	Growth, Migration, Invasion	(Lu et al., 2022)
Endometrial cancer	Upregulation	Oncogene	MCTP1-AS1/miR-650/SMAD7 (TGF-β/SMAD)	Proliferation, Migration, Invasion, EMT	(Gao et al., 2021)
Testicular seminoma	Downregulation	Tumor suppressor	miR-650/TOP2A	Proliferation, Migration, Diagnostic markers	(Wang et al., 2019)
CLL	Upregulation	Oncogene	miR-650/NDRG2/p53	Apoptosis, Binet staging, Overall survival	(Yang et al., 2018)
	Upregulation	Tumor suppressor	miR-650/CDK1, ING4, EBF3	Proliferation	(Mraz et al., 2012)
DLBCL	Downregulation	/	miR-650/TRAF5/NF-κB	Apoptosis, Diagnostic markers	(Li et al., 2022a)
Oral squamous cell carcinoma	Upregulation	Oncogene	circ_0049396/miR-650/NEFH	Malignant phenotype	(Yuan et al., 2023)
Thyroid cancer	Upregulation	Oncogene	miR-650/PPP2CA	Migration, Invasion	(Orlandella et al., 2019)
Osteosarcoma	/	Oncogene	miR-650/ING4/NF-κB	Transcription, Secretion	(Yun et al., 2015)

### 3.1 Colorectal cancer

Colorectal cancer (CRC) is the third most commonly diagnosed cancer worldwide. Although the incidence rate in high-income economies has decreased among the average-age population, it has increased in emerging economies and among young adults (aged <50 years) globally (Eng et al., 2024). In colorectal cancer (CRC), miR-650 functions through both direct targeting of tumor suppressors and indirect regulation via competing endogenous RNA (ceRNA) networks. A consistent finding is miR-650-mediated suppression of multiple tumor suppressors. For instance, miR-650 directly targets ING4 to activate ERK/p38 MAPK signaling, promoting proliferation, migration, and epithelial-mesenchymal transition (EMT) (You et al., 2018). It also downregulates NDRG2 via CpG island hypermethylation (Feng et al., 2011) and inhibits KISS1 to induce apoptosis and overcome 5-fluorouracil resistance (Valizadeh et al., 2023). In parallel, miR-650 activity is tightly modulated by lncRNAs acting as ceRNA sponges. Oncogenic lncRNAs such as DICER1-AS1 and MIR155HG competitively bind miR-650, derepressing targets like MAPK1 and ANXA2, thereby driving CRC progression and drug resistance (Li et al., 2023; Zhou L. et al., 2022). Conversely, tumor-suppressive lncRNAs like MIR22HG can sponge miR-650 and restore expression of downstream targets such as BTK and PDK4 (Liao et al., 2019). Additionally, miR-150-5p and miR-650 may synergistically regulate NF-κB-related genes, though the precise interplay requires further investigation (Slattery et al., 2018). Collectively, these studies illustrate that miR-650s role in CRC is highly context-dependent, governed by the balance between upstream ceRNA regulators and downstream target availability.

### 3.2 Lung cancer

Global estimates indicate that lung cancer was the most common cancer in 2022, with nearly 2.5 million new cases, accounting for one-eighth of global cancers (Leiter et al., 2023). In non-small cell lung cancer (NSCLC) and lung adenocarcinoma (LAD), miR-650 predominantly acts as an oncogene. Overexpression of miR-650 directly suppresses ING4, leading to Wnt-1/β-catenin pathway activation, enhanced proliferation and invasion, and poor prognosis (Tang et al., 2019). This same miR-650/ING4 axis also contributes to docetaxel resistance by modulating Bcl-2/Bax expression (Huang et al., 2013). Beyond direct targeting, ceRNA regulation is evident: the lncRNA CARD8-AS1 exerts tumor-suppressive effects by sponging miR-650 and upregulating Bax (Ji et al., 2022). An interesting genetic mechanism involves a single nucleotide variant (rs277646) that alters alternative polyadenylation of the NIT2 3′UTR. The T allele shortens the 3′UTR, eliminating a miR-650 binding site and allowing NIT2 to escape repression, which promotes LAD cell proliferation and increases cancer risk (Xu et al., 2024). Emerging evidence suggests that epitranscriptomic modifications, including RNA methylation, may influence miRNA biogenesis, stability, and target accessibility. However, whether such modifications directly regulate miR-650 maturation, activity, or target selection remains largely unknown. These findings reveal that miR-650s impact in lung cancer is shaped not only by ceRNA networks but also by genetic variation affecting miRNA-mRNA interactions.

### 3.3 Gastric cancer

Gastric cancer (GC) remains a major clinical challenge, with nearly 1 million new cases and 650,000 deaths each year (Sundar et al., 2025). Clarifying how non-coding RNA such as miR-650 function in tumors may support therapeutic development. In gastric cancer (GC), miR-650 is generally upregulated and functions as an oncogene. It directly targets ING4 to relieve growth inhibition (Zhang et al., 2010), and its transcription is driven by PBX1, which is upregulated in *Helicobacter* pylori-infected tissues; miR-650 then suppresses LATS2 (a Hippo pathway core component), promoting proliferation, migration, and invasion while inhibiting apoptosis (Liu J. et al., 2021). Upstream, several lncRNAs act as ceRNA sponges to modulate miR-650 activity. For example, DICER1-AS1 and linc01105 can sponge miR-650, thereby upregulating targets like CSR1 or TCEA3 and restraining GC progression (An et al., 2021; Zhang et al., 2025). Thus, in GC, miR-650 serves as a downstream effector of both transcription factors and ceRNA regulators, and its oncogenic function is consistently linked to the suppression of tumor suppressors such as ING4 and LATS2.

### 3.4 Melanoma

Melanoma is a highly aggressive tumor with a strong propensity for distant metastasis. Metastatic melanoma is often resistant to available therapies (Long et al., 2023). Defining the mechanisms underlying melanoma is important for developing treatments. In cutaneous melanoma, miR-650 exhibits dual roles depending on the regulatory network context. On one hand, miR-650 directly targets ING4, and its overexpression promotes metastasis by relieving ING4-mediated inhibition (Chen et al., 2024). On the other hand, when sponged by the lncRNA ZFPM2-AS1, miR-650s suppression of NOTCH1 is relieved, leading to NOTCH pathway activation and enhanced cell proliferation and migration-in this context, miR-650 acts as a tumor suppressor (Liu W. et al., 2021). In drug resistance, the lncRNA POU3F3 sponges miR-650, upregulating MGMT and conferring dacarbazine resistance (Wu et al., 2021). These observations highlight that miR-650s functional polarity in melanoma is dictated by the competing endogenous RNA landscape and the specific target gene engaged.

### 3.5 Breast cancer

Breast cancer is the most common cancer among women worldwide. In 2022, there were more than 2.3 million newly diagnosed patients and mortality accounted for 14.1% of all cancers, second only to lung cancer (Gompel and Simcock, 2026). Targeted therapy remains an active area of research in the field of breast cancer treatment, and the mechanisms involving miR-650 may be important. In breast cancer, miR-650 is frequently overexpressed, often due to amplification of the 22q11.2 region, and promotes aggressive phenotypes. Key targets include STAT5A, which when suppressed by miR-650 drives EMT and correlates with poor prognosis in poorly differentiated tumors (López-Huerta and Fuentes-Pananá, 2020). Additionally, miR-650 targets both ING4 and NDRG2, facilitating EMT, invasion, and cancer stem cell properties (Lango-Chavarría et al., 2017). Similar to observations in colorectal, gastric, and lung cancers, miR-650 promotes breast cancer progression mainly through regulation of tumor suppressive targets and EMT-associated pathways. However, breast cancer presents a distinct regulatory context characterized by hormone-related signaling and STAT5A-associated mechanisms. These findings indicate that although miR-650 converges on common malignant processes across cancers, the dominant regulatory pathways are shaped by cancer-specific molecular environments.

### 3.6 Glioma

Among central nervous system tumors, glioma is the most common and aggressive malignant tumor of the brain and has the highest mortality rate of all brain malignancies (Bing et al., 2016). Previous studies indicate that miR-650 is strongly correlated with glioma proliferation, migration, invasion and prognosis (Su et al., 2022). In glioma, miR-650 is consistently upregulated and serves as an independent prognostic marker, with higher expression correlating with advanced WHO grade, lower Karnofsky Performance Status, and shorter overall survival (Sun et al., 2013). Mechanistically, NF-κB1 directly transactivates miR-650, which then targets RERG; this disrupts the RERG-PHLPP2 tumor-suppressive complex, activating AKT/ERK/NF-κB signaling and promoting proliferation, migration, invasion, EMT, and autophagy while inhibiting apoptosis (Jin et al., 2020). These data position miR-650 as a key downstream effector of inflammatory signaling in glioma, though further studies are needed to validate its therapeutic potential.

### 3.7 Hepatocellular carcinoma

Hepatocellular carcinoma (HCC) is the sixth most common cancer globally and the third leading cause of cancer death, after lung and colorectal cancer (Llovet et al., 2024). Non-coding RNAs have been a hot topic in recent years, and their mechanisms in hepatocellular carcinoma (HCC) have been reported. In hepatocellular carcinoma (HCC), the role of miR-650 is strikingly context-dependent, with opposing findings reported. In some settings, miR-650 acts as a tumor suppressor: its downregulation in urine serves as a non-invasive diagnostic biomarker, potentially through targeting TRAF4 (Abdalla and Haj-Ahmad, 2012). Conversely, multiple studies support an oncogenic role. miR-650 is upregulated in HCC tissues, where it promotes proliferation, migration, invasion, and EMT by directly inhibiting SOCS3 and activating JAK/STAT3 signaling; this upregulation can be induced by environmental carcinogens such as benzo[a]pyrene (Ge et al., 2021). This study show specific environmental carcinogens may influence miR-650-associated regulatory networks. However, whether this mechanism represents a general response to environmental stressors or is restricted to specific carcinogenic exposures remains unclear. Additionally, Axin1 negatively regulates miR-650, and its loss contributes to miR-650-mediated oncogenesis (Qin et al., 2020). Interestingly, the anesthetic remifentanil can upregulate the lncRNA NBR2, which sponges miR-650, relieving suppression of TIMP3 and inhibiting HCC progression (Liang and Ke, 2022). These findings suggest that the clinical application of miR-650 requires a comprehensive analysis of the specific molecular environment and etiological background, as well as an in-depth exploration of its dynamic regulatory network, in order to provide a reliable basis for the precise diagnosis and treatment of HCC as well as risk stratification.

### 3.8 Prostate cancer and reproductive system tumors

In prostate cancer, miR-650 is upregulated and promotes proliferation and migration through axes involving LINC01002/miR-650/FLNA and miR-650/CSR1 (Qian et al., 2023; Zuo et al., 2015). In ovarian cancer, however, miR-650 is downregulated and suppresses tumor growth by targeting KLF12 (Lu et al., 2022). In endometrial cancer, miR-650 is upregulated via reduced sponging by MCTP1-AS1, leading to SMAD7 suppression and TGF-β/SMAD pathway activation, which drives EMT (Gao et al., 2021). In testicular seminoma, miR-650 is downregulated and predicted to target TOP2A, with potential involvement in PI3K/AKT and Wnt/β-catenin pathways (Wang et al., 2019). When comparing tissues of origin, these observations raise the possibility that tumors directly associated with reproduction (testicular seminoma and ovarian cancer) might exhibit distinct features in miR-650 expression relative to other tissue types. However, given the limited research in this area, it remains unclear whether such differences, if any, are attributable to as-yet-unidentified mechanisms or pathways, and further investigation is warranted.

### 3.9 Hematological malignancies

Hematological malignancies are malignancies that originate in the hematopoietic system and mainly affect the blood, bone marrow, lymph nodes and spleen (Garbayo et al., 2024). These malignancies are commonly classified into leukemias, lymphomas and multiple myeloma (Manickasamy et al., 2024; Zhang H. et al., 2024). In chronic lymphocytic leukemia (CLL), miR-650 presents contradictory prognostic associations. One study reports that miR-650 is upregulated, targets NDRG2 to suppress p53-mediated apoptosis, and correlates with advanced Binet stage and shorter survival (Yang et al., 2018). Conversely, another study finds that miR-650 expression is linked to immunoglobulin λ light chain V2 family rearrangement and that high miR-650 predicts better prognosis, as it inhibits B-cell proliferation by targeting CDK1, ING4, and EBF3 (Mraz et al., 2012). These discrepancies may reflect subtype-specific heterogeneity. In diffuse large B-cell lymphoma, bioinformatic analysis suggests miR-650 may target TRAF5 and affect NF-κB signaling (Li C. et al., 2022). Different subtypes of hematological malignancies may indeed have significant, as yet undiscovered, differences in the expression and function of miR-650. Tailoring therapeutic strategies to different tumor subtypes may be key to overcoming these diseases, but further research is needed. In the future, the introduction of single-cell RNA sequencing technology capable of identifying the expression patterns of miR-650 in specific cells and revealing the interactions between target cells in complex tissues, as well as CyTOF technology that can perform high-dimensional phenotypic analysis of immune cell populations and their signaling states, will help to gain a more comprehensive understanding of the regulatory mechanism of miR-650 in the disease microenvironment.

### 3.10 Others

In oral squamous cell carcinoma, circ_0049396 is downregulated, leading to increased miR-650 and subsequent suppression of NEFH, which promotes malignant phenotypes (Yuan et al., 2023). In anaplastic thyroid cancer, miR-650 targets PPP2CA to enhance migration and invasion without affecting proliferation (Orlandella et al., 2019). In osteosarcoma, miR-650 targets ING4 to activate NF-κB and increase IL-6 secretion upon IL-1β stimulation, revealing a role in inflammatory responses (Yun et al., 2015). Collectively, these studies reinforce the recurring theme that miR-650s oncogenic or tumor-suppressive actions are dictated by its specific downstream targets and the upstream regulatory RNAs that control its availability.

**FIGURE 2:**
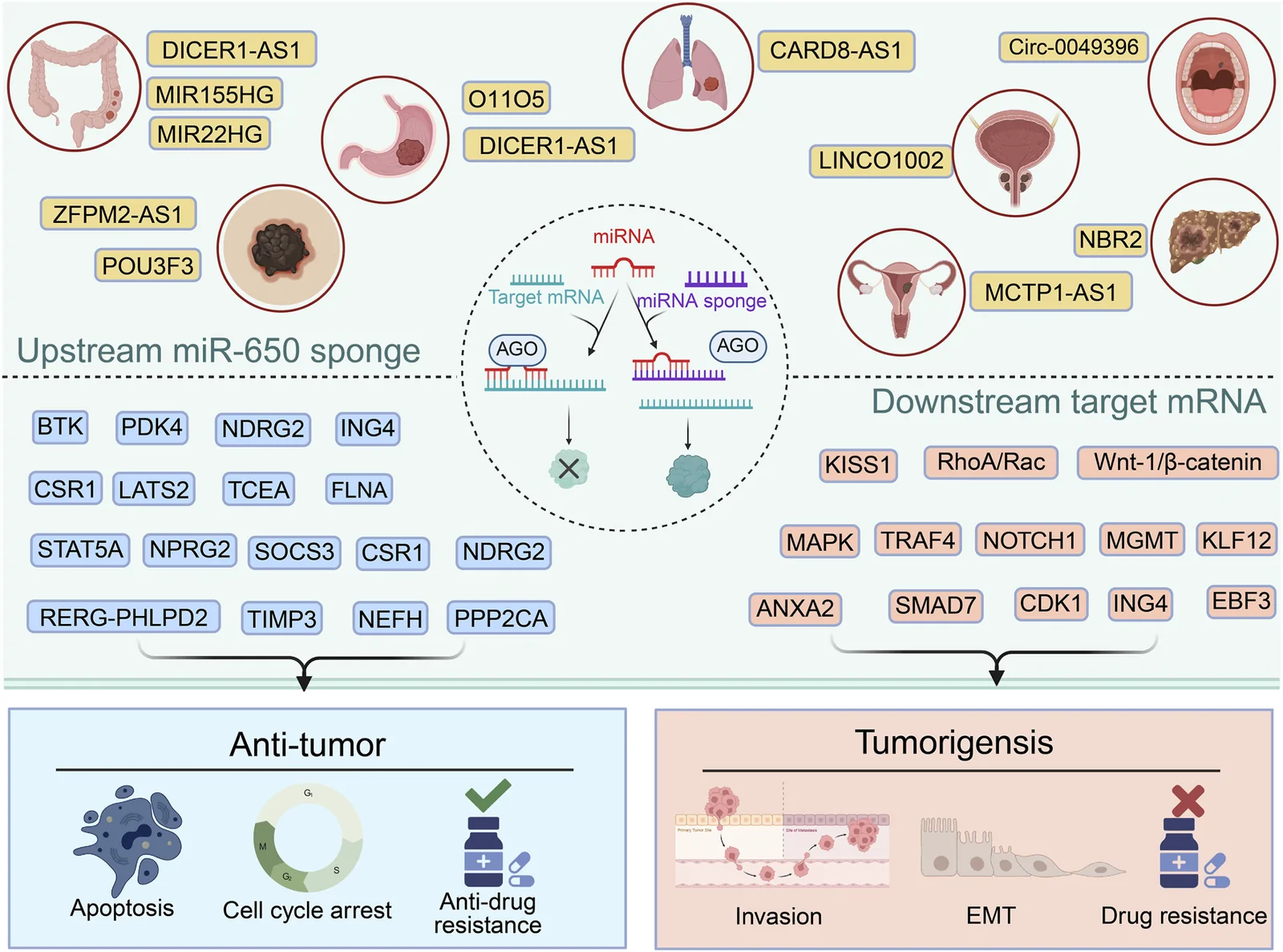
miR-650 regulates malignant tumor-related processes through the ceRNA-miR-650 -target mRNA axis. The upstream ceRNA (long non-coding RNA and circular RNA) can sponge miR-650 and relieve its inhibitory effect on the downstream mRNA-related pathways. Thus, it regulates biological behaviors such as drug resistance, apoptosis, cell cycle, EMT, and invasion.

miR-650, as a non-coding RNA that is broadly involved in tumor progression, mainly inhibits gene expression at the post-transcriptional level. Whether miR-650 promotes cancer progression therefore depends on the function of its target genes. The ceRNA-miR-650-target mRNA is the most extensive regulatory mechanism of miR-650. On one hand, the expression of miR-650 is strictly controlled by upstream regulatory networks, which include ceRNAs that sponge miR-650, such as DICER1-AS1, MIR155HG, MIR22HG, ZFPM2-AS1, POU3F3, NBR2, MCTP1-AS1 and circ_0049396. On the other hand, miR-650 can directly target and inhibit multiple key downstream genes, including ING4, NDRG2, KISS1, LATS2, RERG, SOCS3, SMAD7, PPP2CA and NEFH, thereby regulating signaling pathways such as Wnt/β-catenin, NF-κB, AKT/ERK, JAK/STAT3, Hippo and TGF-β/SMAD, and thus regulating biological behaviors such as cell proliferation, apoptosis, epithelial-mesenchymal transition (EMT), migration, invasion. In addition, transcription factors that directly bind to the promoter region, such as PBX1 and NF-κB1; genetic variations that affect the length of the 3′UTR, such as the APA event caused by rs277646, also participate in the regulatory network of miR-650 (Figure 2). Notably, the role of miR-650 in different cancers is tissue-specific: In different cancers, even within the same type of cancer, due to heterogeneity, miR-650 plays different roles. This complex regulatory network suggests that miR-650 and its upstream/downstream molecules have the potential to serve as diagnostic markers, therapeutic targets and prognostic indicators for many cancers. However, the fine-tuned regulatory mechanisms need to be further investigated for different cancer types and even different subtypes of the same cancer.

## 4 The functions and mechanisms of miR-650 in non-neoplastic diseases

**FIGURE 3:**
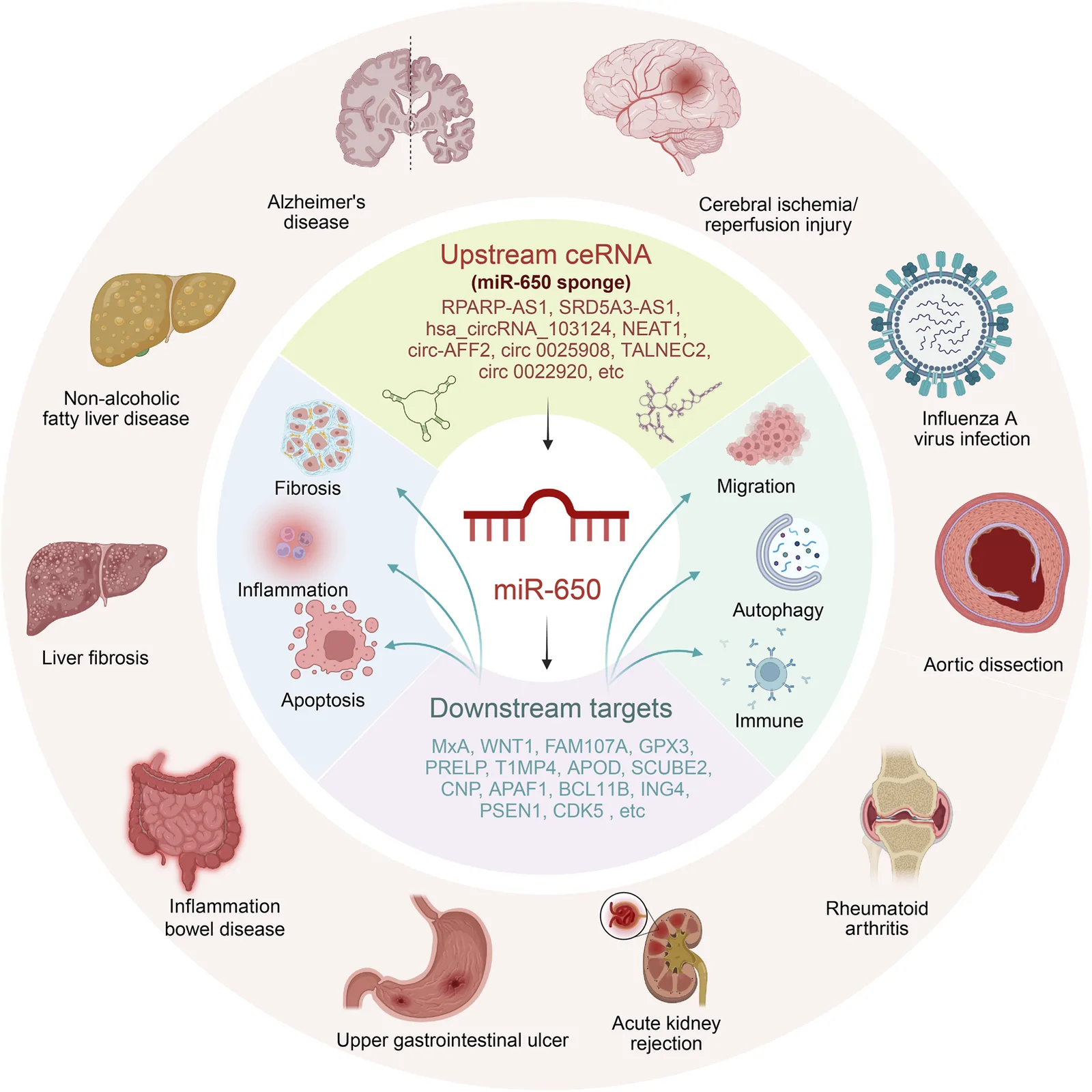
miR-650 regulates non-tumor diseases through the ceRNA-miR-650-target mRNA axis. miR-650 is associated with upstream ceRNAs and downstream target mRNAs via the ceRNA-miR-650-mRNA axis in non-malignant diseases. It influences various biological processes, including fibrosis, inflammation, apoptosis, migration, and autophagy.

Beyond its regulatory roles in malignant tumors, miR-650 contributes to the development and progression of various non-neoplastic diseases (Table 2). Non-neoplastic diseases involving the regulation of miR-650 include nonalcoholic fatty liver disease, inflammatory bowel disease, liver fibrosis, rheumatoid arthritis, acute kidney rejection, Alzheimer’s disease, cerebral ischemia/reperfusion injury and so on (Figure 3). These diseases are often characterized by high incidence, chronic progression, high disability rates, and a lack of effective targeted therapies, imposing a heavy burden on global public health. For example, the global prevalence of non-alcoholic fatty liver disease exceeds 30%, and it can progress to liver fibrosis, cirrhosis, and even liver cancer (Amini-Salehi et al., 2024). The incidence of inflammatory bowel disease continues to increase among young and middle-aged adults (Ng et al., 2017). And the disease of Alzheimer’s disease, which is the most common neurodegenerative disease, still lacks drugs that can reverse its course (Lane et al., 2018). Therefore, detailed investigation of the molecular regulatory networks of these diseases and the discovery of new diagnostic markers and therapeutic targets are of great clinical translational value.

**TABLE 2:** Expression and regulation of miR-650 in non-tumor diseases.

Disease type	miR-650 expression	Prognostic value	Regulatory axis	Function/Role	Reference
NAFLD	Upregulation	Poor	RPARP-AS1/miR-650/LATS2	Participate in the pathogenesis of NAFLD	(Hany et al., 2022)
	Upregulation	/	RPARP-AS1, SRD5A3-AS1/miR-650/AMOTL2, NF2	Promote NAFLD development via Hippo signaling	(Matboli et al., 2021)
Liver fibrosis	Upregulation	/	/	Distinguish between liver fibrosis, liver cirrhosis and liver cancer	(Gibriel et al., 2022)
CD	Downregulation	Favorable	hsa_circRNA_103124/miR-650/AKT2	Promote M1 macrophage polarization and the inflammatory microenvironment	(Yin et al., 2021)
	Downregulation	Favorable	hsa_circRNA_103124/miR-650/AKT2	Restoring miR-650 can inhibit M1 polarization, alleviate inflammation and protect the barrier	(Yin et al., 2023)
UC	Upregulation	Poor	miR-650/NLRP6	Promote apoptosis of intestinal epithelial cells and intensify inflammation	(Xu et al., 2019)
	Upregulation	/	NEAT1/miR-650/SLC6A14, IRAK3	/	(Li et al., 2022b)
Upper gastrointestinal ulcer	Upregulation	/	/	Infection marker	(Lario et al., 2012)
Acute kidney rejection	Upregulation	Poor	miR-650/BCL11B	Promote the apoptosis of glomerular endothelial cells, the release of inflammatory factors and the chemotaxis of macrophages; aggravate acute rejection reactions	(Jin et al., 2017)
RA	/	Favorable	circ-AFF2/miR-650/CNP	Promote the proliferation, migration, invasion and inflammation of synovial cells	(Qu et al., 2021)
	Downregulation	Favorable	circ_0025908/miR-650/SCUBE2	Inhibit the abnormal proliferation, migration and inflammation of synovial cells	(Wang et al., 2022)
AD	Upregulation (Brain tissue)	Favorable	miR-650/CDK5	Reduce Aβ plaques and neuronal damage; exert a protective effect	(Lin et al., 2023)
	/	/	/	/	(Prendecki et al., 2019)
Cerebral ischemia/reperfusion	Upregulation	Poor	TALNEC2/miR-650/APAF1	Aggravates apoptosis of nerve cells and brain damage	(Cao et al., 2021)
IAV	Downregulation	/	PRR/miR-650/MxA	Promote the antiviral state	(Pichulik et al., 2016)
Articular cartilage injury	Downregulation	Favorable	miR-650/WNT1	Promote the proliferation of chondrocytes; inhibit their apoptosis and inflammation; play a protective role against articular cartilage injury	(Liu et al., 2024)
COPD	Downregulation	/	miR-650/FAM107A, GPX3, PRELP, TIMP4, APOD	/	(Wu et al., 2024)
Aortic dissection	Upregulation	Poor	Circ_0022920/miR-650/RF1, TGFβR1	Promote the proliferation, migration and phenotypic transformation of vascular smooth muscle cells; accelerate the progression of aortic dissection	(Huang et al., 2023)

### 4.1 Non-alcoholic fatty liver disease

Non-alcoholic fatty liver disease (NAFLD) has an estimated global prevalence of 25% and is an important risk factor for cirrhosis and liver cancer (Powell et al., 2021). In non-alcoholic fatty liver disease (NAFLD), miR-650 is upregulated and contributes to fibrosis progression. It directly targets LATS2, a core Hippo pathway component, leading to activation of Hedgehog and Hippo signaling, hepatic stellate cell activation, and increased secretion of IL-6 and TGF-β (Hany et al., 2022). Upstream, the lncRNAs RPARP-AS1 and SRD5A3-AS1 negatively regulate miR-650, and their downregulation in NAFLD permits miR-650 overexpression (Matboli et al., 2021). Importantly, the probiotic Mutaflor® can upregulate RPARP-AS1, thereby sponging miR-650 and attenuating fibrotic signaling (Hany et al., 2022). This provides a proof-of-concept that modulating the ceRNA network upstream of miR-650 may offer a therapeutic strategy for NAFLD, though clinical translation remains to be established.

### 4.2 Liver fibrosis

Liver fibrosis is prevalent worldwide. Several factors, including obesity, non-alcoholic fatty liver disease and hepatitis viruses, may contribute to its development and progression. If the cause of the disease is not eliminated, it may develop towards liver cirrhosis or even liver cancer (Ginès et al., 2021). In HCV-related liver fibrosis, plasma miR-650 is upregulated in early fibrosis stages but significantly downregulated in hepatocellular carcinoma, showing high diagnostic accuracy (AUC = 0.8067) for distinguishing HCC from non-HCC. Bioinformatics predicts that miR-650 targets fibrosis-related genes such as TRAF4, Rac1, AXL, NCOA1, and KLF6, suggesting its potential as a non-invasive staging biomarker (Gibriel et al., 2022). However, direct mechanistic studies in liver fibrosis are still limited.

### 4.3 Crohn’s disease

Crohn’s disease is an inflammatory bowel disease of unclear etiology that involves immune dysregulation in genetically susceptible individuals (Dolinger et al., 2024). Identifying disease-specific immune targets may therefore help control its occurrence and development. Some studies have found that miR-650 plays a role in the inflammatory response of Crohn’s disease, which might be the direction of our further research. In two studies by Yin et al. on Crohn’s disease, it was revealed that the hsa_circRNA_103124/miR-650/AKT2 axis plays a central pathogenic role. By sponging miR-650, hsa_circRNA_103124 relieves AKT2 repression, leading to a multifaceted cascade: promotion of M1 macrophage polarization and inflammation via TLR4/NF-κB, suppression of autophagy via TSC1 inhibition, and disruption of intestinal epithelial barrier integrity (Yin et al., 2023; Yin et al., 2021). This integrated pathway highlights how a single ceRNA-miR-650-target axis coordinates complex inflammatory and tissue-damaging processes, making it a promising therapeutic target for CD.

### 4.4 Ulcerative colitis

Ulcerative colitis (UC), is a lifelong inflammatory bowel disease associated with defects in the intestinal epithelial barrier, dysregulation of the intestinal microbiota and immune response (Le Berre et al., 2023). Identifying specific targets that mediate the occurrence of inflammation might be a possible direction to delay its development in the future. In ulcerative colitis (UC), miR-650 is upregulated in inflamed mucosa and directly targets NLRP6, promoting intestinal epithelial apoptosis and aggravating colitis; inhibition of miR-650 alleviates inflammatory symptoms (Xu et al., 2019). Additionally, the lncRNA NEAT1 may sponge miR-650, regulating downstream targets SLC6A14 and IRAK3 (Li et al., 2022b). These findings position miR-650 as a pro-inflammatory mediator in UC, and its targeting could represent a novel therapeutic approach.

### 4.5 Upper gastrointestinal ulcer

Upper gastrointestinal ulcers are closely related to *Helicobacter pylori* (*H. pylori*) infection and related inflammatory responses (Dovjak, 2017). In H. pylori-associated chronic active gastritis, miR-650 is upregulated approximately 10-fold in antral mucosa, irrespective of duodenal ulcer presence, and its expression correlates with gastritis activity. miR-650 alone shows moderate diagnostic accuracy for *H. pylori* infection (AUC = 0.8659), and combined with miR-96 improves performance (Lario et al., 2012). Future studies should prioritize the establishment of disease-relevant animal models, CRISPR-based manipulation of miR-650 expression, single-cell sequencing approaches to identify cell-specific functions, and prospective clinical cohorts to determine the diagnostic and therapeutic value of miR-650.

### 4.6 Acute kidney rejection

Transplantation can be life-saving for patients with end-stage organ failure. However, immune rejection remains a major challenge in xenotransplantation (Zhou et al., 2022b). In acute renal allograft rejection, miR-650 is upregulated and targets BCL11B, suppressing cell proliferation, inducing apoptosis, and promoting inflammatory cytokine release and macrophage chemotaxis (Jin et al., 2017). This suggests miR-650 contributes to the rejection process, but its utility as a therapeutic immune target requires validation in larger cohorts.

### 4.7 Rheumatoid arthritis

Rheumatoid arthritis (RA), is a chronic systemic autoimmune disease in which inflammation control remains central to disease management (Jang et al., 2022). In rheumatoid arthritis (RA), two circular RNAs, circ-AFF2 and circ_0025908, are upregulated in synovial tissues and fibroblasts, where they sponge miR-650. This relieves miR-650-mediated suppression of targets CNP and SCUBE2, respectively, promoting fibroblast proliferation, migration, invasion, and inflammation while inhibiting apoptosis (Qu et al., 2021; Wang et al., 2022). These studies converge on a common theme: upregulated circRNAs act as miR-650 sponges, derepressing pro-inflammatory targets and driving RA pathogenesis. Targeting these circRNA/miR-650 axes may offer new therapeutic avenues.

### 4.8 Alzheimer’s disease

Alzheimer’s disease (AD), is the leading cause of dementia in older adults and has been studied extensively. However, to date, there is still no universally effective treatment. Continued investigation of its pathogenesis may provide a theoretical basis for future therapeutic development (Scheltens et al., 2021). In Alzheimer’s disease (AD), miR-650 exhibits tissue-specific expression patterns. In brain tissue, processing of pri-miR-650 is dysregulated, leading to increased mature miR-650, which targets CDK5 and reduces Aβ plaque formation and neuronal damage, suggesting a protective role (Lin et al., 2023). However, in plasma, miR-650 levels are decreased in AD patients and correlate with worse cognitive function, possibly due to altered transport or oxidative stress (Prendecki et al., 2019). Together, these studies indicate that miR-650 exhibits tissue-specific expression patterns in Alzheimer’s disease. The increased miR-650 observed in brain tissues and the decreased circulating miR-650 detected in plasma may reflect different biological processes rather than contradictory findings. Brain-derived miR-650 may represent intracellular regulatory responses within neural cells, whereas circulating miR-650 may be influenced by extracellular transport, blood-brain barrier changes, and systemic responses. In addition, the cellular composition of brain tissue may contribute to these differences, as neurons, glial cells, and immune-related cells may exhibit distinct miR-650 expression profiles. Disease stage and pathological progression may further influence miR-650 distribution and function. Therefore, future studies should integrate tissue-specific sampling, single-cell sequencing, and spatial transcriptomic approaches to clarify the cellular origin and functional consequences of miR-650 dysregulation in Alzheimer’s disease.

### 4.9 Cerebral ischemia/reperfusion injury

In cerebral ischemia/reperfusion (I/R) injury, the lncRNA TALNEC2 sponges miR-650, reducing its availability, and miR-650 directly targets APAF1 (apoptotic protease activating factor 1). Overexpression of miR-650 protects neurons from oxygen-glucose deprivation/reoxygenation-induced apoptosis, whereas TALNEC2 exacerbates injury by sequestering miR-650 (Cao et al., 2021). Thus, miR-650 acts as a protective factor in this pathway, and strategies to restore its activity could be neuroprotective.

### 4.10 Influenza

The annual seasonal influenza epidemic caused by the influenza virus causes a substantial global disease burden (Uyeki et al., 2022). In influenza A virus (IAV) infection of monocyte-derived dendritic cells, pattern recognition receptor signaling downregulates miR-650, relieving suppression of its target MxA, an antiviral effector, thereby promoting an antiviral state (Pichulik et al., 2016). Future investigations should move beyond descriptive associations and focus on establishing causal relationships and translational relevance. Functional validation using miR-650 knockout or overexpression models, including CRISPR/Cas9-based approaches and miRNA mimic/inhibitor experiments, will be essential to determine whether miR-650 directly drives disease phenotypes. Rescue experiments restoring specific downstream targets will further clarify the molecular mechanisms involved.

### 4.11 Articular cartilage injury

In osteoarthritis, miR-650 is downregulated, and its overexpression protects chondrocytes by targeting WNT1, promoting proliferation, inhibiting apoptosis, and counteracting IL-1β-induced inflammation (Liu et al., 2024). Although current evidence in osteoarthritis remains limited, the biological functions of miR-650 are broadly consistent with those reported in other inflammatory diseases. Similar to rheumatoid arthritis, Crohn’s disease, and ulcerative colitis, miR-650 participates in regulating inflammatory responses, apoptosis, and tissue remodeling through disease-specific downstream targets. Notably, while the direct target genes differ among diseases (WNT1 in osteoarthritis, CNP and SCUBE2 in rheumatoid arthritis, AKT2 in Crohn’s disease, and NLRP6 in ulcerative colitis), these pathways converge on common pathological processes including inflammatory signaling, cell survival, and tissue repair. This observation suggests that miR-650 may function as a conserved regulator of inflammatory homeostasis across multiple non-neoplastic diseases despite disease-specific molecular contexts. Future comparative studies should determine whether these apparently distinct regulatory pathways ultimately converge on common inflammatory signaling networks, thereby facilitating the development of broader miR-650-targeted therapeutic strategies.

### 4.12 Chronic obstructive pulmonary disease

In a study of chronic obstructive pulmonary disease (COPD), bioinformatic analysis identified five potential miR-650-mRNA pairs (involving FAM107A, GPX3, PRELP, TIMP4, and APOD) that may be associated with glucocorticoid therapy in COPD (Wu et al., 2024). However, mechanistic and translational studies are lacking, and this remains a preliminary finding.

### 4.13 Aortic dissection

miR-650 also contributes to cardiovascular disease. In aortic dissection, circ_0022920 is downregulated, reducing its capacity to sponge miR-650. Subsequently, elevated miR-650 targets IRF1, reducing TGFβR1 expression and promoting vascular smooth muscle cell proliferation, migration, and phenotype switching from contractile to synthetic (Huang et al., 2023). This axis represents a potential therapeutic target for maintaining vascular integrity.

Studies of miR-650 in non-neoplastic diseases indicate recurring regulatory features despite marked differences among diseases. Similar to its role in cancer, miR-650 is frequently embedded within ceRNA networks, in which lncRNAs and circRNAs regulate its availability and subsequently influence downstream target genes. Across different inflammatory, metabolic, and degenerative disorders, several signaling pathways repeatedly emerge as major regulatory nodes. For example, NF-κB signaling is involved in inflammatory activation in diseases such as Crohn’s disease and rheumatoid arthritis, whereas Hippo-related pathways contribute to metabolic dysfunction and fibrosis in liver diseases. PI3K/AKT and TGF-β signaling further connect miR-650 regulation with cell survival, immune responses, and tissue remodeling. Although the specific targets of miR-650 differ among diseases, including AKT2, NLRP6, CNP, SCUBE2, LATS2, and WNT1, these targets converge on common pathological processes such as inflammation, apoptosis regulation, immune modulation, and tissue repair. Therefore, miR-650 may represent a common regulatory node linking diverse disease-associated pathways rather than functioning through isolated disease-specific mechanisms. Future studies integrating multi-disease datasets and comparative molecular analyzes may help determine whether shared miR-650 regulatory signatures exist across different non-neoplastic disorders.

## 5 Shared regulatory networks and molecular hubs of miR-650

Although miR-650 has been studied across diverse diseases, several recurring regulatory features are evident. In most reported studies, miR-650 functions as a component of ceRNA-mediated regulatory networks, where lncRNAs and circRNAs regulate miR-650 availability through competitive binding. Subsequently, miR-650 modulates downstream target genes involved in major pathological processes, including proliferation, apoptosis, inflammation, fibrosis, and immune regulation.

Although miR-650 has been associated with multiple signaling pathways, these pathways do not necessarily represent independent regulatory events. miR-650-mediated regulation instead converges on several interconnected signaling networks that control cell proliferation, survival, inflammation, and tissue remodeling.

The NF-κB pathway is a major downstream network influenced by miR-650. Through regulation of targets such as ING4 and inflammation-related regulators, miR-650 can modulate inflammatory responses and malignant phenotypes (Jin et al., 2020). The Hippo pathway is another important regulatory axis, mainly through LATS2 suppression, linking miR-650 with YAP/TAZ-dependent proliferation and tissue remodeling (Liu J. et al., 2021). In addition, PI3K/AKT signaling provides a common survival pathway through regulation of AKT-related molecules, while Wnt/β-catenin signaling contributes to stemness and EMT (Richie, 2020).

These pathways also exhibit extensive crosstalk. For example, PI3K/AKT signaling can interact with NF-κB activation to promote cell survival and inflammatory responses, whereas Hippo and Wnt pathways jointly regulate proliferation and stem-like properties (Guo et al., 2024; Zhang C. et al., 2024). Therefore, the biological effects of miR-650 likely arise from coordinated modulation of signaling networks rather than activation of a single pathway.

## 6 Comparison of miR-650 with other multifunctional miRNAs in diseases

The functional versatility of miR-650 can be assessed by comparison with several well-characterized miRNAs involved in both cancer and non-neoplastic diseases (Table 3). miR-155 is a immunomodulatory miRNA whose effects vary by cell type and disease setting frequently upregulated in multiple cancers, including oral squamous cell carcinoma and lung cancer, where it promotes tumor progression and therapy resistance (Gulei et al., 2019). It also plays important roles in inflammatory and autoimmune diseases such as rheumatoid arthritis and inflammatory bowel disease by regulating macrophage activation and cytokine production (Xu et al., 2022). What’s more, miR-21 is one of the most extensively studied oncomiRs and is broadly overexpressed in many cancers, including breast, lung, colorectal cancer, and glioblastoma. It promotes tumorigenesis mainly by suppressing tumor suppressors such as PTEN and PDCD4 and activating PI3K/AKT signaling (Hashemi et al., 2023). It is also a key regulator of fibrotic processes in multiple organs (Huang et al., 2015). Next, miR-146a mainly functions as a negative regulator of inflammation and is involved in immune homeostasis and autoimmune diseases such as rheumatoid arthritis (Mortazavi-Jahromi et al., 2020). In cancer, it generally shows tumor-suppressive activity, although context-dependent roles have been reported (Iacona and Lutz, 2019). Moreover, miR-34a is a canonical p53-dependent tumor suppressor widely involved in cancer inhibition (Kalfert et al., 2020). It is also implicated in Alzheimer’s disease and fibrotic disorders, where it contributes to neuronal dysfunction and tissue remodeling (Chua and Tang, 2019). Lastly, miR-221 is a well-known oncogenic miRNA that promotes tumor progression in hepatocellular carcinoma, glioma, and other cancers through PTEN/PI3K-AKT signaling, and is also involved in metabolic and fibrotic diseases (Di Martino et al., 2022).

**TABLE 3:** Comparison of miR-650 with other well-characterized multifunctional miRNAs.

miRNA	Role in cancer	Role in non-neoplastic diseases	Classic target genes and pathways
miR-155	Oncogene	Inflammatory diseases, Autoimmune disorders, Organ fibrosis	SOCS1, TP53INP1/ NF-κB, JAK-STAT
miR-21	Oncogene	Organ fibrosis, Cardiovascular diseases	PTEN, PDCD4/ PI3K-AKT
miR-146a	Dual role	Inflammation and immune regulation, Neuroinflammation	TRAF6, IRAK1/ NF-κB
miR-34a	Tumor suppressor	Neurodegenerative diseases, Organ fibrosis	CDK4, BCL2, SIRT1/ p53
miR-221	Oncogene	Metabolic diseases, Liver fibrosis	PTEN, p27/ PI3K-AKT

Compared with these miRNAs, miR-650 has similarly diverse functions but shows more pronounced functional polarity among cancer types, acting as either an oncogene or tumor suppressor depending on the cell type, tissue, and available targets. In addition, miR-650 is regulated by an extensive ceRNA network involving multiple lncRNAs and circRNAs, and has been reported in association with copy-number alterations in the 22q11.2 region in certain cancers. Functionally, miR-650 is implicated in diverse pathological processes including tumor progression, inflammation, fibrosis, and neurodegeneration. Thus, miR-650 is a multifunctional regulatory miRNA with roles that vary by disease, cell type, and target availability across both malignant and non-neoplastic diseases.

## 7 Challenges and opportunities for clinical translation of miR-650

Although miR-650 has potential as a diagnostic biomarker and therapeutic target, several challenges must be addressed before clinical application. Unlike conventional protein biomarkers, the biological effect of miR-650 is highly dependent on tissue origin, cellular composition, disease subtype, and target availability. The same expression alteration may generate different outcomes depending on tissue origin, cellular composition, disease subtype, and downstream target availability. Therefore, miR-650 expression alone may not be sufficient for accurate diagnosis or therapeutic decision-making. Integrating miR-650 expression with target gene signatures, molecular classification, and patient-specific characteristics may improve its clinical utility.

Therapeutic modulation of miR-650 requires disease-specific strategies. miR-650 mimics may restore tumor-suppressive functions in diseases in which it has tumor-suppressive activity, whereas anti-miR-650 approaches may be considered when miR-650 promotes pathological processes. However, efficient and selective delivery remains a major limitation (Bravo-Vázquez et al., 2023). Emerging delivery platforms, including lipid nanoparticles, polymer-based carriers, and engineered exosomes, may improve miRNA stability, cellular uptake, and tissue targeting (Wang et al., 2025). Nevertheless, challenges related to biodistribution, manufacturing consistency, and long-term safety remain unresolved. Lipid nanoparticles are attractive because of their ability to encapsulate nucleic acids, protect miRNAs from enzymatic degradation, and facilitate intracellular delivery. Nevertheless, challenges including preferential accumulation in specific organs, limited tissue specificity, and potential immune activation remain important barriers for broader clinical application (Haghighi et al., 2024). Polymer-based nanoparticles offer tunable tunable physicochemical properties, surface modification, and controlled release characteristics. However, their clinical translation requires further optimization regarding biodegradability, toxicity, and long-term biological compatibility (Rananaware et al., 2026). Exosome-based delivery is another potential strategy because exosomes naturally mediate intercellular RNA communication and exhibit favorable biocompatibility. Engineered exosomes may improve tissue targeting and reduced toxicity compared with synthetic carriers. However, issues related to large-scale production, cargo loading efficiency, purification standardization, and quality control remain unresolved (Corraliza-Gomez et al., 2026).

Off-target effects are another major concern. Because miRNAs regulate multiple transcripts simultaneously, systemic modulation of miR-650 may influence unintended signaling pathways in non-target tissues. Therapeutic development should therefore incorporate precise delivery systems and comprehensive transcriptomic evaluation to minimize unexpected biological effects (Diener et al., 2022). Immunogenicity is also an important safety consideration. Exogenous RNA molecules or delivery materials may activate innate immune responses through pattern recognition receptors, potentially affecting therapeutic efficacy and safety. Development of miR-650-based therapies should therefore include systematic evaluation of immune activation, inflammatory responses, and long-term toxicity (Samad and Kamaroddin, 2023).

Although several miRNA-based therapeutic approaches have entered clinical investigation, most primarily evaluate safety, pharmacokinetics, and preliminary efficacy (Seyhan, 2024). miR-650-specific therapeutic strategies are still largely restricted to preclinical studies, and substantial work is needed to establish their clinical feasibility.

Most current evidence regarding miR-650 is derived from cell-based experiments, animal models, or retrospective clinical samples. Although these studies provide mechanistic insight, prospective clinical validation remains limited. Prospective studies should use standardized detection methods and large patient cohorts to determine whether miR-650-based biomarkers or therapies provide additional value beyond existing clinical parameters.

## 8 Conclusion and perspective

miR-650 is a conserved, functionally versatile microRNA that regulates in malignant tumors and non-neoplastic diseases. Its reported mechanisms follow two main patterns. First, miR-650 directly binds the 3′ untranslated region (UTR) of target mRNAs and represses downstream genes, including ING4, NDRG2, LATS2, RERG, SOCS3, SMAD7, AKT2, NLRP6, BCL11B, CNP, SCUBE2, CDK5, APAF1, WNT1, and IRF1. Through these targets, miR-650 affects cell proliferation, apoptosis, autophagy, EMT, inflammation, fibrosis, and immune responses. Second, miR-650 functions as a node in ceRNA networks and can be sponged by upstream lncRNAs or circRNAs, including DICER1-AS1, MIR155HG, MIR22HG, ZFPM2-AS1, POU3F3, NBR2, MCTP1-AS1, circ_0049396, RPARP-AS1, NEAT1, hsa_circRNA_103124, circ-AFF2, circ_0025908, TALNEC2, and circ_0022920. These interactions relieve repression of downstream target mRNAs and form ceRNA regulatory networks.

In cancer, miR-650 has primarily been reported to act as an oncogenic factor in gastric cancer, breast cancer, glioma, prostate cancer, endometrial cancer, oral squamous cell carcinoma, thyroid cancer, and osteosarcoma by promoting proliferation, migration, invasion, EMT, and chemoresistance. By contrast, it has been reported to act as a tumor suppressor in ovarian cancer and testicular seminoma. In colorectal cancer, lung cancer, melanoma, HCC, and CLL, miR-650 may have dual or context-dependent effects. In non-neoplastic diseases, miR-650 is implicated in nonalcoholic fatty liver disease, inflammatory bowel disease, rheumatoid arthritis, Alzheimer’s disease, cerebral ischemia/reperfusion injury, aortic disease, and related conditions. Across these settings, it can exert proinflammatory, profibrotic, proapoptotic, or protective effects. Notably, shared miR-650-targeting axes, including ING4, LATS2, and AKT2, recur across multiple diseases, suggesting broad regulatory potential. Tissue-specific expression and disease-dependent effects, such as contradictory prognostic associations in CLL, indicate that any intervention will require disease- and tissue-specific evaluation.

Several limitations remain. Most mechanistic studies rely on cell or animal models, and clinical translational evidence is still limited. Upstream transcriptional regulation, epigenetic modification, and genetic variation, including APA events involving miR-650, have not been fully characterized. In addition, miR-650 expression heterogeneity across disease subtypes requires further study. Future studies should apply complementary technologies to define miR-650 regulation more precisely. Single-cell RNA sequencing may help identify cell-specific miR-650 expression patterns and determining whether altered miR-650 levels originate from tumor cells, immune populations, stromal cells, or other cellular compartments. Spatial transcriptomic approaches may reveal the tissue distribution and microenvironmental interactions of miR-650-associated regulatory networks. Because miR-650 functions vary by cell type and disease, spatial information may clarify how cellular localization influences its downstream effects. Recent advances in spatial miRNA profiling support the feasibility of resolving miRNA regulation at tissue level. In addition, CRISPR-based approaches may enable more precise functional validation of miR-650. Compared with conventional miRNA mimics or inhibitors, CRISPR-mediated knockout, activation, or interference strategies may enable evaluation under more physiological conditions of miR-650 function and its therapeutic potential. Artificial intelligence and machine learning approaches may improve miR-650 target discovery and clinical stratification by integrating sequence information, RNA structure, multi-omics datasets, and patient-specific molecular profiles. These computational approaches may help identify disease-specific miR-650 regulatory networks and improve prediction of patients who may benefit from miR-650-based interventions. Integrating single-cell technologies, spatial omics, genome editing, and artificial intelligence may move miR-650 research beyond descriptive associations toward mechanistic studies and carefully validated therapeutic applications.
